# Chalcones and Dihydrochalcones Augment TRAIL-Mediated Apoptosis in Prostate Cancer Cells

**DOI:** 10.3390/molecules15085336

**Published:** 2010-08-04

**Authors:** Ewelina Szliszka, Zenon P. Czuba, Bogdan Mazur, Andrzej Paradysz, Wojciech Krol

**Affiliations:** 1 Department of Microbiology and Immunology, Medical University of Silesia in Katowice, Jordana 19, 41 808 Zabrze, Poland; E-Mails: eszliszka@sum.edu.pl (E.S.); zczuba@sum.edu.pl (Z.P.C.); bmazur@sum.edu.pl (B.M.); 2 Department of Urology, Medical University of Silesia in Katowice, 3-go Maja 13, 41 800 Zabrze, Poland; E-Mail: parady@poczta.onet.pl (A.P.)

**Keywords:** chalcones and dihydrochalcones, TRAIL, apoptosis, chemoprevention, prostate cancer

## Abstract

Chalcones and dihydrochalcones exhibit chemopreventive and antitumor activity. TRAIL (tumor necrosis factor-related apoptosis-inducing ligand) is a natural endogenous anticancer agent. We examined the cytotoxic and apoptotic effect of chalcones and dihydrochalcones on TRAIL-mediated apoptosis in LNCaP prostate cancer cells. The cytotoxicity was evaluated by the MTT and LDH assays. The apoptosis was detected using annexin V-FITC by flow cytometry and fluorescence microscopy. The ΔΨm was evaluated using DePsipher staining by fluorescence microscopy. Our study showed that two tested chalcones (chalcone and 2’,6’dihydroxy-4’-methoxychalcone) and three dihydrochalcones (2’,6’-dihydroxy-4’4-dimethoxydihydrochalcone, 2’,6’-dihydroxy-4’-methoxydihydro- chalcone, and 2’,4’,6’-trihydroxydihydrochalcone, called phloretin) markedly augmented TRAIL-induced apoptosis and cytotoxicity in LNCaP cells and confirmed the significant role of chalcones in chemoprevention of prostate cancer.

## 1. Introduction

Chalcones and dihydrochalcones are a family of bicyclic compounds, which are precursors for flavonoid biosynthesis in plants. They are defined by the presence of two aromatic rings linked by a three carbon bridge that is unsaturated in chalcones and saturated in dihydrochalcones. Chalcones and dihydochalcones exert multiple biological activities, including antiinflammatory, antioxidant and anticancer properties [1,2,3,4,5,6]. 

Prostate cancer is one of the most commonly diagnosed cancers in men, and the second leading cause of cancer deaths in the EU and the USA [7]. Several naturally derived polyphenolic compounds have been studied in prostate cancer in an attempt to identify effective preventative therapies for this disease. Chemoprevention represent a promising approach in prostate cancer research, in which natural or synthetic agents are used to prevent this malignant disease [8]. Chalcones and dihydrochalcones are cancer-preventive food components found in a human diet rich in fruits and vegetables [9,10]. The *in vitro* studies demonstrated that chalcones inhibit the proliferation of prostate cancer cells by inducing apoptosis and blocking cell cycle progression [11,12,13,14,15].

**Figure 1:**
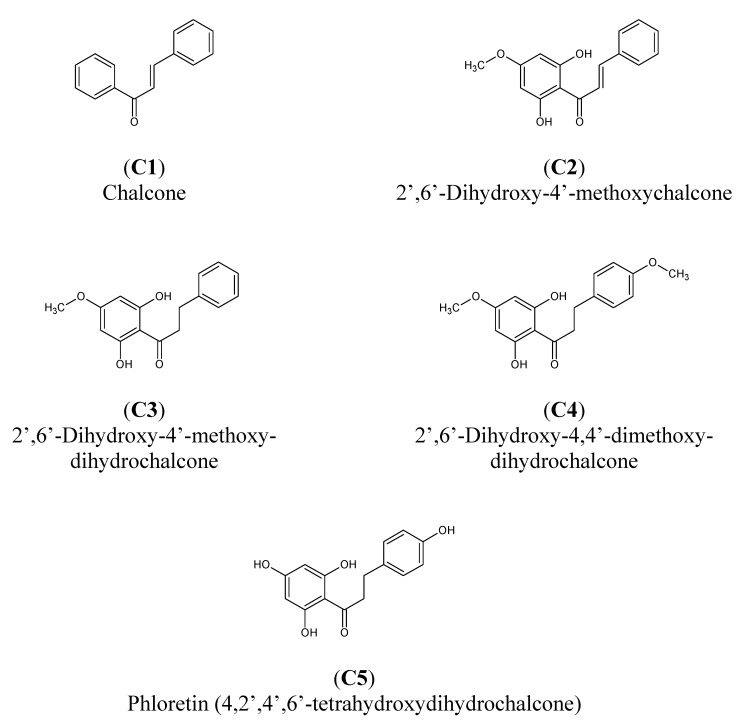
Chemical structures of the studied chalcones and dihydrochalcones.

In this work we investigated the cytotoxic and apoptotic effect of chalcones: (**C1**) chalcone, (**C2**) 2’6’-dihydroxy-4’-methoxychalcone and dihydrochalcones: (**C3**) 2’6’-dihydroxy-4’-methoxydihydro-chalcone, (**C4**) 2’,6’-dihydroxy-4,4’-dimetoxydihydrochalcone, (**C5**) 4,2’,4’,6’-tetrahydroxydihydro-chalcone (named phloretin) in combination with TRAIL on prostate cancer cells. Figure 1 presents the structures of chalcones and dihydrochalcones used in this study. 

Tumor necrosis factor related apoptosis inducing ligand (TRAIL) induces apoptosis selectively in cancer cells with no toxicity against normal tissues. Soluble or expressed on lymphocytes T, NK cells, dendritic cells, neutrophils, monocytes or macrophages molecules TRAIL play an important role in immune surveillance and defense mechanisms against tumor cells. TRAIL mediates programmed death through its interaction with the death receptor TRAIL-R1 (DR4) and/or TRAIL-R2 (DR5) [16,17]. However, some tumor cells are resistant to TRAIL-mediated cytotoxicity. The decreased expression of death receptors TRAIL-R1 and TRAIL-R2 or other proapoptotic proteins as well as the increased expression of antiapoptotic proteins in cancer cells are involved in TRAIL-resistance [18]. We and others showed that TRAIL-resistant prostate cancer cells can be sensitized by dietary polyphenols [19,20,21]. 

In our previous study we reported that some chalcones (chalcone, licochalcone-A, isobavachalcone, xanthohumol and butein) enhance TRAIL-induced apoptosis in LNCaP prostate cancer cells [22]. The present findings show for the first time that in addition to chalcones (chalcone and 2’6’-dihydroxy-4’-metoxychalcone), dihydrochalcones (2’6’-dihydroxy-4’-metoxydihydrochalcone, 2’6’-dihydroxy-4,4’-dimetoxydihydrochalcone and 4,2’,4’,6’-tetrahydroxydihydrochalcone, called phloretin) also markedly augmented TRAIL-mediated apoptosis and cytotoxicity in prostate cancer cells. The obtained results confirmed the significant role of chalcones and dihydrochalcones in prostate cancer chemoprevention through sensitization of cancer cells to TRAIL-induced programmed death.

## 2. Results and Discussion

### 2.1. Cytotoxic and apoptotic effects of chalcones and dihydrochalcones in prostate cancer cells

Chalcones and dihydrochalcones are naturally occurring compounds belonging to the flavonoid family and widely investigated for their chemopreventive and antitumor activity in various therapeutic areas [9]. Isoliquirtigenin induced cell cycle arrest in DU145 and MLL prostate cancer cells [12]. Licochalcone-A blocked cell cycle in PC3 prostate cancer cells [13]. Xanthohumol and desmethylxanthohumol inhibited proliferation of DU145 and PC3 cells [14]. Isobavachalcone mediated apoptosis in PC3 cells [15]. Chalcone arrested cell cycle progression and promoted apoptosis in T24 and HT1376 bladder cancer cells or MCF7 and MDAMB231 breast cancer cells [23,24]. Cryptocaryone, a dihydrochalcone, induces apoptosis in PC3 prostate cancer cells [25].

We showed that treatment of LNCaP prostate cancer cells with chalcones and dihydrochalcones hydroxylated or methoxylated in the positions 4, 2’, 4’, 6’ inhibits cell proliferation by induced cytotoxicity and apoptosis. 

**Figure 2:**
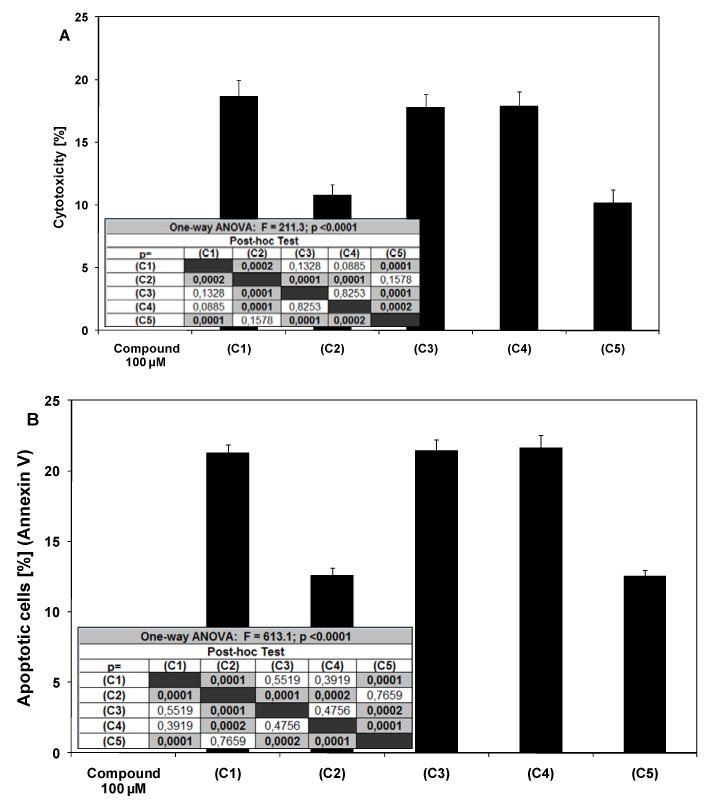
Cytotoxic and apoptotic effects of chalcones and dihydrochalcones on LNCaP prostate cancer cells. The cancer cells were incubated for 48 hours with compounds C1–C5 at the concentrations of 100 μM. The values represent mean ±SD of three independent experiments performed in quadruplicate (n = 12) for cytototoxicity, or in duplicate (n = 6) for apoptosis (p < 0.05). **(A)** Cytotoxic activity of chalcones and dihydrochalcones in LNCaP cells. The percentage of cell death was measured by MTT cytotoxicity assay. **(B)** Apoptotic activity of chalcones and dihydrochalcones in LNCaP cells. Detection of apoptotic cell death by annexin V-FITC staining using flow cytometry.

The cancer cells were incubated for 48 hours with chalcone, 2’,6’-dihydroxy-4’-methoxychalcone, 2’,6’-dihydroxy-4’-methoxydihydrochalcone, 2’,6’-dihydroxy-4,4’-dimethoxydihydrochalcone and phloretin at the concentrations of 50 μM and 100 μM. The chalcones and dihydrochalcones induced cytotoxicity in LNCaP cells in a dose dependent manner. The cytotoxic effect of tested compounds on LNCaP cells were 5.65 ± 1.15%–12.53 ± 0.84% cell death for 50 μM and 10.15 ± 1.06%–18.65 ± 1.33% cell death for 100 μM (Figure 2A). The apoptotic activities of chalcones and dihydrochalcone at the concentration of 100 μM were 21.26 ± 0.60% for chalcone, 12.58 ± 0.53% for 2’,6’-dihydroxy-4’-methoxychalcone, 21.43 ± 0.82% for 2’,6’-dihydroxy-4’-methoxy-dihydrochalcone, 21.63 ± 0.89% for 2’,6’-dihydroxy-4,4’-dimethoxydihydrochalcone, 12.50 ± 0.48% for phloretin. The annexin V assay revealed apoptotic cells exposed to chalcones and dihydrochalcones (Figure 2B). The most potent cytotoxic and apoptotic activity against LNCaP cells exhibited chalcone, 2’,6’-dihydroxy-4’-methoxydihydrochalcone and 2’,6’-dihydroxy-4,4’dimethoxy-dihydrochalcone.

### 2.2. Cytotoxic and apoptotic effects of TRAIL in prostate cancer cells

**Figure 3:**
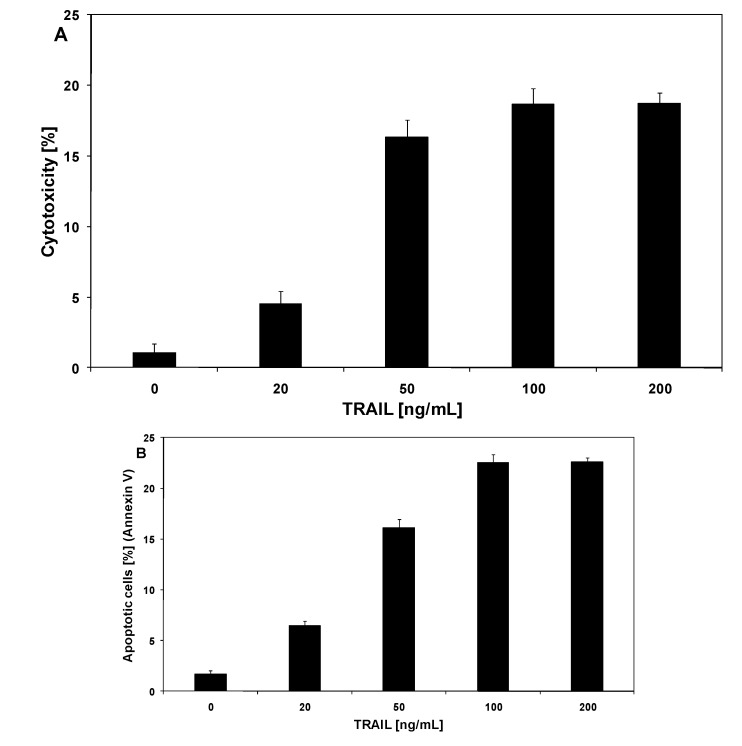
Cytotoxic and apoptotic effect of TRAIL on LNCaP prostate cancer cells. The cancer cells were incubated for 48 hours with TRAIL at the concentrations of 20–200 ng/mL. The values represent mean ±SD of three independent experiments performed in quadruplicate (n = 12) for cytototoxicity, or in duplicate (n = 6) for apoptosis (p < 0.05). **(A)** Cytotoxic activity of TRAIL in LNCaP cells. The percentage of cell death was measured by MTT cytotoxicity assay. **(B)** TRAIL-induced apoptosis in LNCaP cells. Detection of apoptotic cell death by annexin V-FITC staining using flow cytometry.

Apoptosis of premalignant or malignant cells represents a protective mechanism against tumor formation and development, since it removes from the body genetically damaged cells induced to proliferate under uncontrolled mitogenic stimuli. TRAIL is a naturally occurring anticancer agent appearing in soluble form or expressed in immune cells. TRAIL mediates *in vitro *and *in vivo *apoptosis in cancer cells [16,17]. Resistance to programmed cell death is a hallmark of cancer, with both loss of proapoptotic signals (decreased expression of death receptors TRAIL-R1 and/or TRAIL-R2) and the gain of the antiapoptotic mechanisms (increased expression of antiapoptotic proteins) contributing to tumorigenesis [18]. We and others have shown that LNCaP prostate cancer cells are resistant to TRAIL-mediated cytotoxicity [20,21,22]. TRAIL induced cytotoxic and apoptotic effects in LNCaP cells in a dose-dependent manner. The cytotoxicity of TRAIL at the concentrations of 20–200 ng/mL after 48 hours’ incubation were 4.51 ± 0.93%–18.75 ± 0.74% cell death (Figure 3A). Figure 3B presents TRAIL induced apoptosis in LNCaP cancer cells determined by annexin V staining followed by flow cytometry. The 48 hours’ exposure to TRAIL increased the percentage of apoptotic cells in a dose-dependent manner to 6.45 ± 0.45%–22.62 ± 0.42%. 

Accumulating data clearly indicate that induction of apoptosis is an important event for chemoprevention of cancer by naturally occurring dietary agents [26]. The role of polyphenols in prostate cancer prevention has been confirmed in previous laboratory and epidemiology studies, however the mechanism of chemopreventive activities by these compounds largely remains unknown [8]. Chalcones constitute an important group of flavonoids which show various biological activities including antioxidant, anticancer and immunomodulatory properties [27,28].

### 2.3. Cytotoxic and apoptotic effects of TRAIL in combination with chalcones and dihydrochalcones in prostate cancer cells

**Figure 4:**
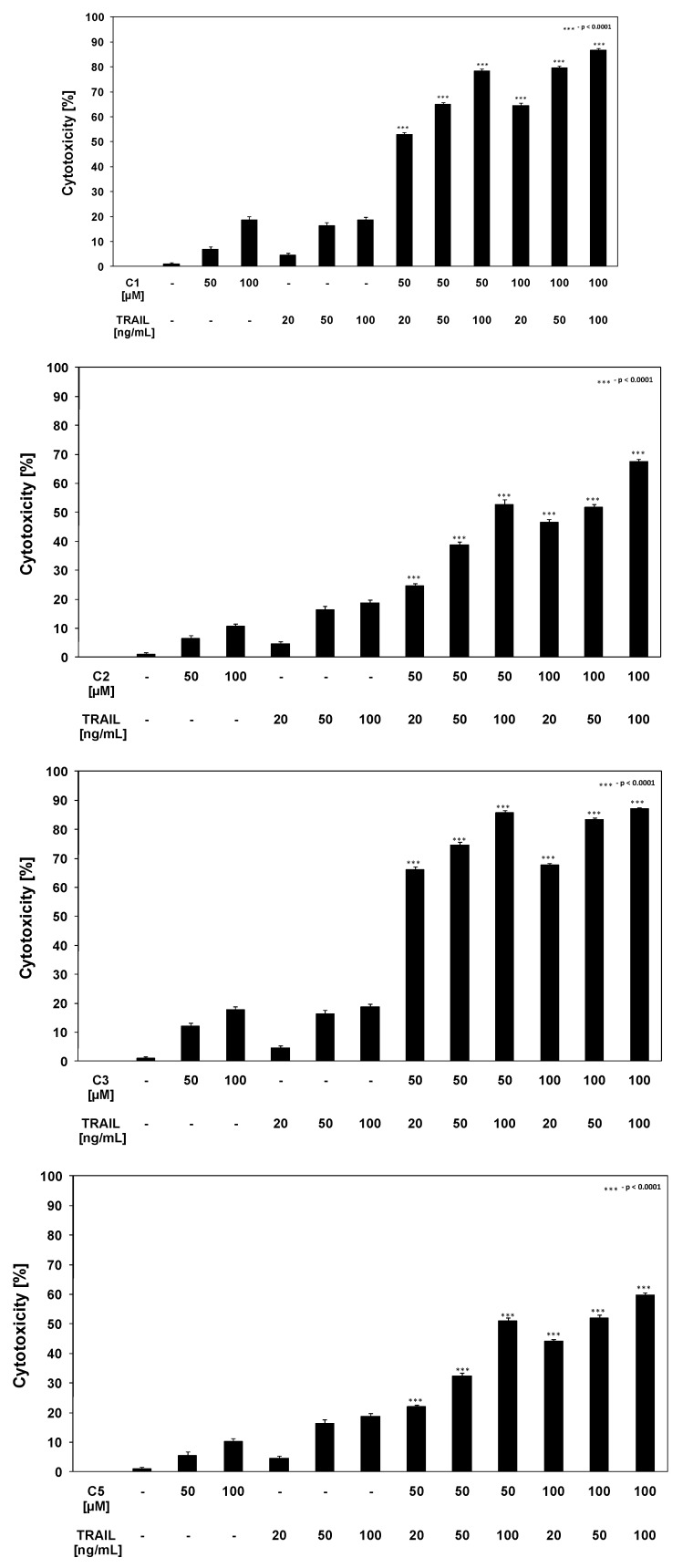
Cytotoxic activity of TRAIL in combination with chalcones and dihydrochalcones in LNCaP prostate cancer cells. The cancer cells were incubated for 48 hours with TRAIL at the concentrations of 20–100 ng/mL and the compounds C1–C5 at the concentrations of 50–100 μM. The percentage of cell death was measured by MTT cytotoxicity assay. The values represent mean ±SD of three independent experiments performed in quadruplicate (n = 12). ^***^ = significantly different from control or TRAIL (p < 0.0001).

TRAIL is a key effector molecule responsible for surveillance against tumor development. Jakobisiak *et al.* confirmed the role of TRAIL in the immune mechanisms of protection against cancer [29]. Immunomodulation through natural substances may be considered as an alternative for prevention of malignant disease. In our previous study we demonstrated that several chalcones (chalcone, licochalcone-A, isobavachalcone, xanthohumol and butein) enhance TRAIL-induced apoptosis in LNCaP prostate cancer cells [22]. Ferguson and Philpott reported about dietary bioactive food components interacting with the immune response and having potential to reduce the risk of cancer [30]. As shown in Figure 2 and Figure 3, chalcones and dihydrochalcones or TRAIL alone induced little apoptotic and cytotoxic effect on LNCaP cells. We then tested chalcones and dihydrochalcones in combination with TRAIL on prostate cancer cells. The cytotoxicity measured by a MTT assay is shown in Figure 4. 

**Figure 5:**
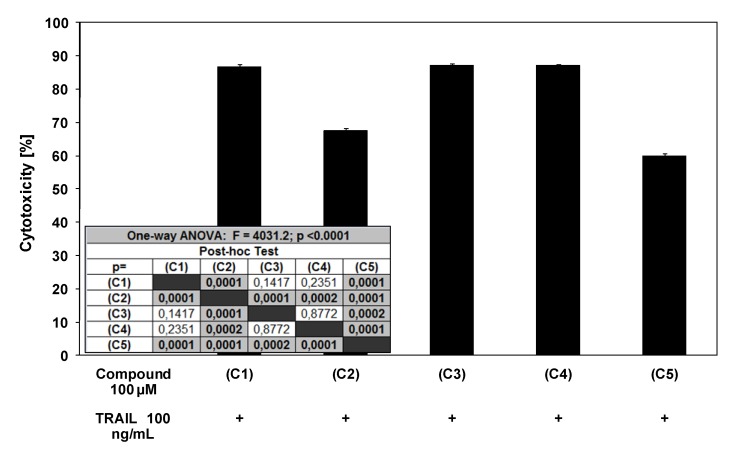
Statistical analysis of cytotoxic activity of TRAIL in combination with chalcones and dihydrochalcones in LNCaP prostate cancer cells. The data are expressed as mean ±SD (n = 12) (p < 0.05).

Chalcones and dihydrochalcones in combination with TRAIL increased the percentage of cell death (52.84 ± 0.83%–86.66 ± 0.88% for chalcone, 24.65 ± 0.82%–67.59 ± 0.76% for 2’,6’-dihydroxy-4’-methoxychalcone, 66.27 ± 0.84%–87.09 ± 0.52% for 2’,6’-dihydroxy-4’-methoxydihydrochalcon, 64.08 ± 0.93%–87.14 ± 0.43% for 2’,6’-dihydroxy-4,4’-dimethoxydihydrochalcone and 22.06 ± 0.59%–59.86 ± 0.85% for phloretin) compared to cytotoxicity of TRAIL and chalcones or dihydrochalcones alone. Figure 5 shows the statistical analysis of cytotoxicity of TRAIL in combination with chalcones or dihydrochalcones.

Tested chalcones, dihydrochalcones and TRAIL induced their cytotoxic effect in cancer cells by the apoptotic pathway, as the necrotic cell death percentage of LNCaP cells examined by Apoptest-FITC and lactate dehydrogenase assay was near 0%.

**Figure 6:**
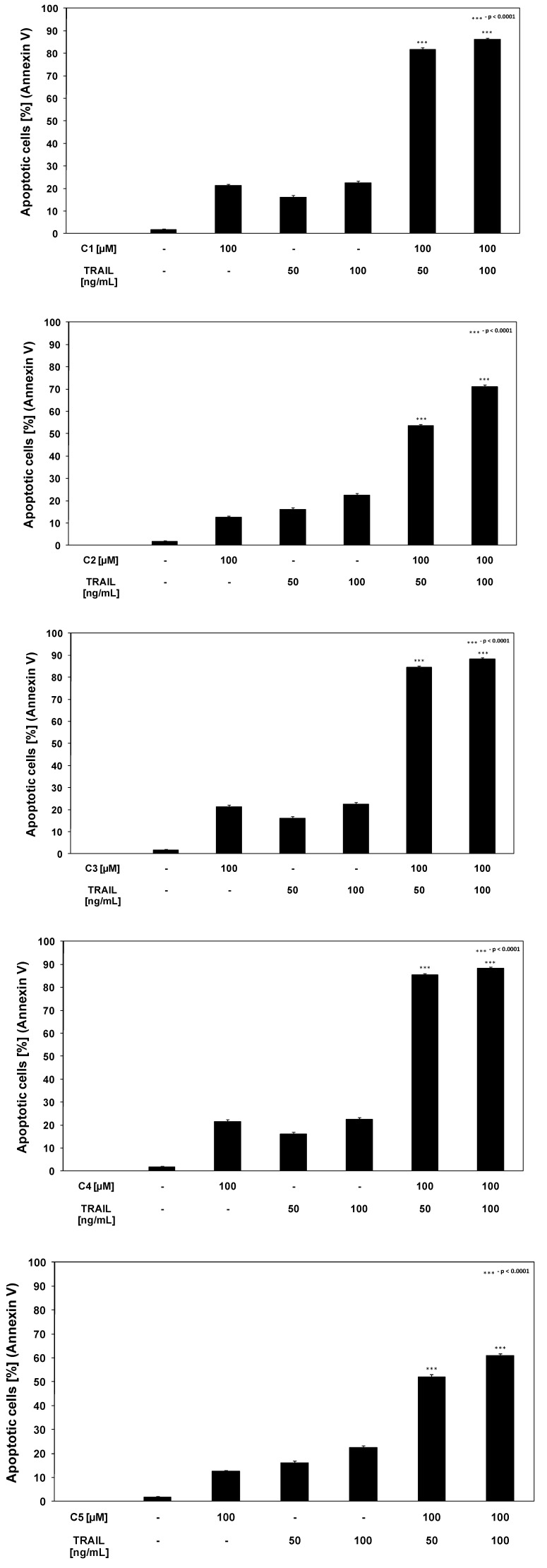
TRAIL induced apoptosis in combination with chalcones and dihydrochalcones in LNCaP prostate cancer cells. The cancer cells were incubated for 48 hours with TRAIL at the concentrations of 50–100 ng/mL and the compounds C1–C5 at the concentrations of 50–100 μM. Detection of apoptotic cell death by annexin V-FITC staining using flow cytometry. The values represent mean ±SD of three independent experiments performed in duplicate (n = 6). ^*** ^= significantly different from control or TRAIL (p < 0.0001).

**Figure 7:**
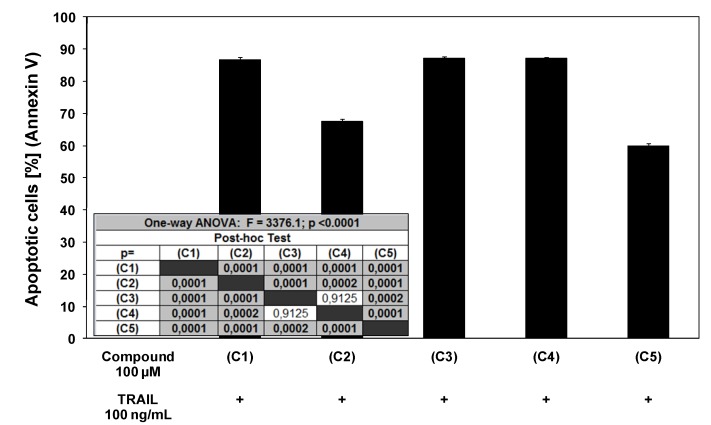
Statistical analysis of apoptotic activity of TRAIL in combination with chalcones and dihydrochalcones in LNCaP prostate cancer cells. The data are expressed as mean ±SD (n = 6) (p < 0.05).

**Figure 8:**
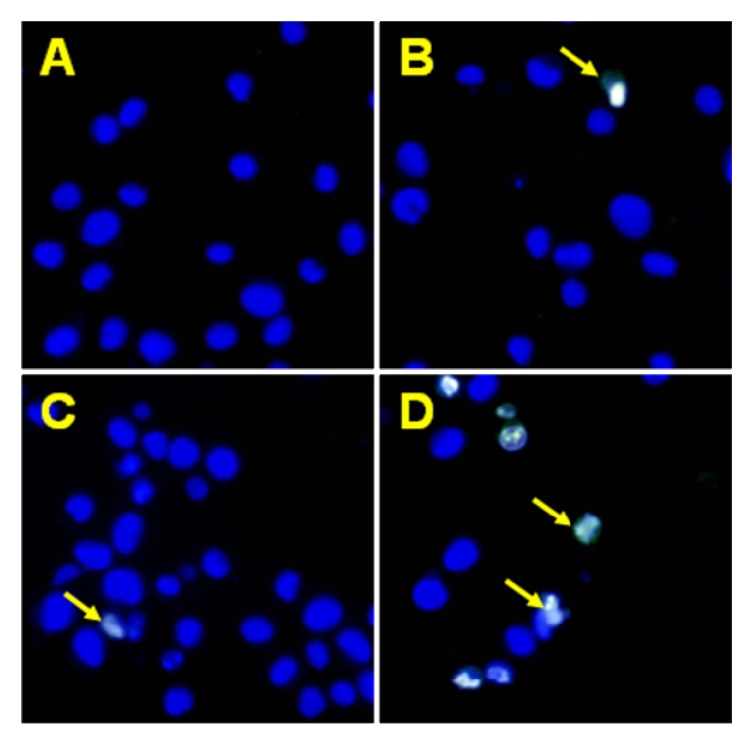
TRAIL in combination with chalcone induced apoptosis in LNCaP prostate cancer cells. The cancer cells were incubated for 48 hours with TRAIL at the concentration of 100 ng/mL and chalcone at the concentration of 100 μM. Detection of apoptotic cell death by annexin V-FITC staining using fluorescent microscopy. The healthy cells (stained with Hoechst 33342) emitted blue fluorescence and apoptotic cells. Apoptotic cells (stained with Annexin V-FITC and Hoechst 33342) emitted green and blue fluorescence (indicated by arrows). **(A)** Control cells, **(B)** cells incubated with TRAIL (100 ng/mL), **(C)** cells incubated with chalcone (100 μM), **(D)** cells incubated with TRAIL (100 ng/mL) and chalcone (100 μM).

We found out that chalcones and dihydrochalcones strongly cooperated with TRAIL to induce apoptosis in LNCaP cells. The percentage of the apoptotic cells after 48 hours’ exposure to 100 ng/mL TRAIL and 100 μM compounds were elevated at 86.23 ± 0.67% for chalcone, at 71.07 ± 0.84% for 2’,6’-dihydroxy-4’-methoxychalcone, at 88.21 ± 0.66% for 2’,6’-dihydroxy-4’-methoxydihydrochalcone, at 88.24 ± 0.64% for 2’,6’-dihydroxy-4,4’-dimethoxydihydrochalcone and at 60.87 ± 0.86% for phloretin (Figure 6). Figure 7 illustrates the statistical analysis of TRAIL-mediated apoptosis in combination with chalcones or dihydrochalcones. The annexin V-FITC staining by fluorescence microscopy (Figure 8) confirmed the apoptotic activity of TRAIL and chalcone cotreatment against LNCaP cells.

**Figure 9:**
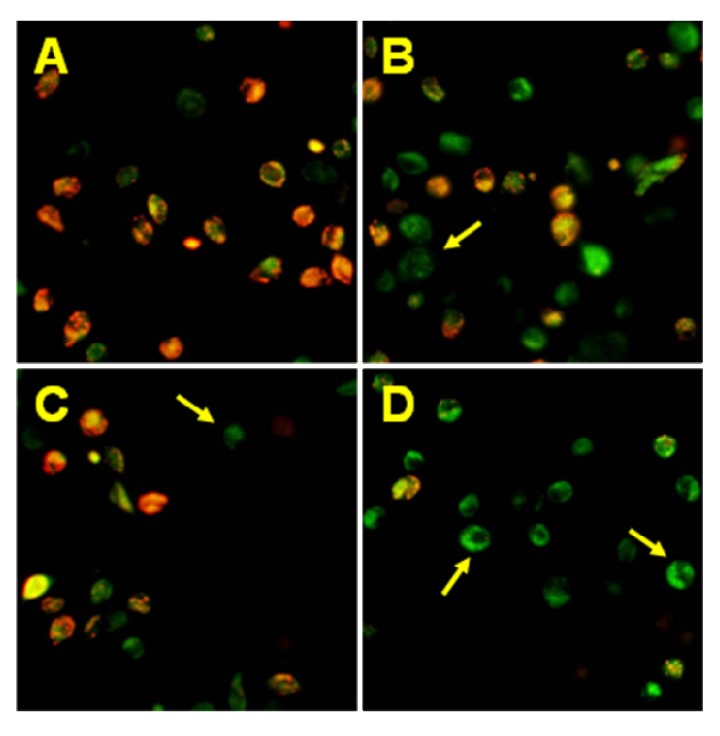
TRAIL in combination with chalcone altered the mitochondrial transmembrane potential (ΔΨm) and induced apoptosis in LNCaP prostate cancer cells. The cancer cells were incubated for 48 hours with TRAIL at the concentration of 100 ng/mL and chalcone at the concentration of 100 μM. DePsipher has the property of aggregation upon membrane polarization forming a red fluorescent compound. If the potential is disturbed, the dye cannot access the transmembrane space and remains or reverts to its green monomeric form. The fluorescence is observed and analyzed by microscope. Apoptotic cells with loss ΔΨm are indicated by arrows. **(A)** Control cells, **(B)** cells incubated with TRAIL (100 ng/mL), **(C)** cells incubated with chalcone (100 μM), **(D)** cells incubated with TRAIL (100 ng/mL) and chalcone (100 μM).

Cellular energy produced during mitochondrial respiration is stored as an electrochemical gradient across the mitochondrial membrane. This accumulation of energy in healthy cells created ΔΨm named mitochondrial transmembrane potential. Changes of ΔΨm in LNCaP cells after cotreatment with TRAIL and chalcone were evaluated using DePsipher staining by fluorescence microscopy (Figure 9). Disruption of ΔΨm has been shown to be one of the first intracellular changes following the onset of apoptosis. DePsipher exhibits potential-dependent accumulation in mitochondria, indicated by a fluorescence emission shift from red to green. The prostate cancer cells with high mitochondrial membrane potential form DePsipher aggregates that are detected by red fluorescence. The cells with low mitochondrial membrane potential DePsipher become a monomer displaying green fluorescence.

Our results demonstrated that chalcones and dihydrochalcones markedly augmented TRAIL-mediated apoptosis in prostate cancer cells. Chalcone, 2’,6’-dihydroxy-4’-methoxydihydrochalcone and 2’,6’-dihydroxy-4,4’-dimethoxydihydrochalcone exhibited the strongest cytotoxic and apoptotic effects in combination with TRAIL against LNCaP cells. In the study the presence of a methoxyl group and probably a hydroxyl group in position 4 of dihydrochalcones is less important, but the presence of a hydroxyl group in position 4’ (*i.e*. phloretin) decreases cytotoxic and apoptotic activities. This suggests the important role of the basic structure of chalcone in cytotoxic and apoptotic reactions. 

In our previous tests chalcone, licochalcone-A, isobavachalcone, xanthohumol and butein enhanced TRAIL-induced apoptosis in LNCaP prostate cancer cells. Xanthohumol alone and in combination with TRAIL was the most active agent in apoptosis induction of cancer cells [22]. Except chalcone there is no evidence of TRAIL cotreatment with other tested compound.

Chalcones and dihydrochalcones restored TRAIL sensitivity in TRAIL-resistant LNCaP cells. Three similar studies with flavokawain B, isoliquiritigenin and butein demonstrated that chalcones synergistically mediated apoptosis in malignant tumor cells with ligand TRAIL principally *via *induction of death receptor TRAIL-R2 (DR5) [31,32,33]. Tang *et al. *showed increased expression of TRAIL-R2 receptor and proapoptotic protein Bim in PC3 prostate cancer cells after cotreatment with TRAIL and flavokawain B and in this way increased percentage of apoptotic cells [31]. Yoshida *et al.* indicated that isoliquiritigenin overcomes TRAIL-resistance in HT29 human colon cancer cells through upregulation of receptor TRAIL-R2 [32]. Kim explained the molecular mechanism by which butein augments TRAIL-mediated apoptosis in U937 human leukemia cells and confirmed the ability of butein to increase expression of receptor TRAIL-R2 and the caspase-3 activation [33]. 

Our study showed the impact of chalcones and dihydrochalcones on the anticancer immune defense through the interaction and modulation of the TRAIL-mediated apoptotic pathway in prostate cancer cells. The findings suggest that chalcones and dihydrochalcones may exert a chemopreventive effect in cooperation with endogenous TRAIL *in vivo*. The TRAIL potential enhancement by chalcones or dihydrochalcones indicated that these compounds can be used in prostate cancer chemoprevention.

## 3. Experimental

### 3.1. Chemicals

#### 3.1.1. Chalcones

The chalcones: chalcone (**C1**), 2’,6’-dihydroxy-4’-methoxychalcone (**C2**), and dihydrochalcones: 2’,6’-dihydroxy-4’-methoxydihydrochalcone (**C3**), 2’,6’-dihydroxy-4,4’-dimethoxydihydrochalcone (**C4**), and phloretin (4,2’,4’,6’-tetrahydroxydihydrochalcone, **C5**) were purchased from Carl Roth GmbH (Karlsruhe, Germany). The tested compounds were dissolved in dimethylsulphoxide (DMSO) to obtain the working concentrations.

#### 3.1.2. TRAIL

Recombinat human TRAIL was purchased from PeproTech Inc. (Rocky Hill, NJ, USA).

### 3.2. Cell culture

The experiments were performed on human hormone-sensitive prostate cancer LNCaP cells (DSMZ - German Collection of Microorganisms and Cell Cultures, Braunschweig, Germany). The cells were maintained in RPMI 1640 medium with 10% fetal bovine serum, 4 mM L-glutamine, 100 U/mL penicillin, and 100 μg/mL streptomycin and were grown in monolayer cultures at the temperature 37 ºC and atmosphere containing 5% CO_2_ [22,34]. Reagents for cell culture were purchased from PAA, The Cell Culture Company (Pasching, Austria).

### 3.3. Cytotoxicity assay

The cytotoxicity was measured by the 3-[4,5-dimethylthiazol-2-yl]-2,5 diphenyltetrazolium (MTT) assay as described [34,35]. The MTT assay is based on the cleavage of the tetrazolium salt MTT to form blue formazan dye by viable cells. The LNCaP cells (2x10^5^/mL) were seeded 48 hours before the experiments in a 96-well plate. Various combinations of chalcones and dihydrochalcones (50 μM and 100 μM) with or without TRAIL (50–200 ng/mL) were added to the cells. After 48 hours the medium was removed, and 20 μL MTT solutions (5 mg/mL) (Sigma Chemical Company, MO, USA) were added to each well for 4 hours. The resulting crystals were dissolved in DMSO. Controls included native cells and medium alone. The spectrophotometric absorbance at 550 nm was measured using a microplate reader (ELx 800, Bio-Tek Instruments Inc., Winooski, VT, USA). The percent cytotoxicity was calculated by the formula: percent cytotoxicity (cell death) = (1-[absorbance of experimental wells/absorbance of control wells]) × 100%. 

### 3.4. Lactate dehydrogenase release assay

Lactate dehydrogenase (LDH) is a stable cytosolic enzyme that is released upon membrane damage in necrotic cells. LDH activity was measured using a commercial cytotoxicity assay kit (Roche Diagnostics GmbH, Mannheim, Germany), in which LDH released in culture supernatants is measured with a coupled enzymatic assay, resulting in conversion of a tetrazolium salt into a red formazan product. The LNCaP cells were treated with various concentrations of chalcones and dihydrochalcones (50 μM and 100 μM) alone and in combination with TRAIL (50–200 ng/mL) for the indicated period of time. The sample solution (supernatant) was removed, and the LDH released from the cells into culture medium was measured. The maximal release was obtained after treating control cells with 1% Triton X-100 (Sigma Chemical Company, St. Louis, MO) for 10 minutes at room temperature [35,36]. The necrotic percentage was expressed using the formula: (sample value/maximal release) × 100%.

### 3.5. Detection of apoptosis by flow cytometry

Apoptosis was measured using flow cytometry to quantify the levels of phosphatidylserine (PS) on the outer membrane of apoptotic cells. Externalized PS on the outer surface of the cytoplasmic membrane becomes labelled by Annexin V-FITC, which has a high affinity for PS-containing phospholipid bilayers. The Annexin V assay was performed using the Apoptotest-FITC Kit (Dako, Glostrup, Denmark). Prostate cancer cell line LNCaP (2 × 10^5^/mL) cells were seeded in 24-well plates for 48 hours and then exposed to chalcones and dihydrochalcones (100 μM) and/or TRAIL (50–100 ng/mL) for 48 hours. After this time cancer cells were washed twice with PBS (phosphate-buffered saline solution) and resuspended in binding buffer (1 mL). The cell suspension (500 μL) was then incubated with Annexin V-FITC (5 μL) and propidium iodide (PI, 10 μL) for 10 minutes at room temperature in the dark. The population of Annexin V-positive cells was evaluated by flow cytometry (BD FACScan, Becton Dickinson Immnunocytometry Systems, San Jose, CA, USA) [21,22,35].

### 3.6. Detection of apoptosis by fluorescence microscopy

Apoptotic cells were quantified by fluorescence microscopy method using the Apoptotic & Necrotic & Healthy Cells Quantification Kit from Biotium, Inc. (Hayward, CA, USA) according to the manufacturer’s instructions [36,37]. The LNCaP cells (2.5 × 10^5^/mL) were seeded 48 hours before the experiments in a 24-well plate. The chalcone (50 μM and 100 μM) with or without TRAIL (100 ng/mL) was added to the cancer cells, and 48 hours later, the cells were washed with PBS and detached from the cell culture wells by trypsin. Next, the LNCaP cells were centrifuged to discard the supernatant, washed with PBS and resuspended in binding buffer (100 μL/sample). A combination of Annexin V-FITC (5 μL), Ethidium Homodimer III (5 μL) and Hoechst 33342 (5 μL) solutions was added to each tube. The samples were incubated at room temperature for 15 minutes in the dark. After staining, the cancer cells were washed with binding buffer, placed on a glass slide and covered with a glass coverslip. The stained cells were observed under a fluorescence inverted microscope IX51 (Olympus, Tokyo, Japan) using filter sets for FITC, TRITC and DAPI. The healthy cells (stained with Hoechst 33342) emitted blue fluorescence, apoptotic cells (stained with Annexin V-FITC and Hoechst 33342) emitted green and blue fluorescence and necrotic cells (stained with Ethidium Homodimer III and Hoechst 33342) emitted red and blue fluorescence. Cancer cells stained with blue, red and green, were dead cells progressing from the apoptotic cell population. The cells were counted and the apoptotic cells were expressed as percentage of total cells.

### 3.7. Evaluation of mitochondrial potential by DePsipher

The DePsipher Kit (R&D Systems, Minneapolis, MN, USA) was used to measure the mitochondrial membrane potential in fluorescence microscopy assay according to the manufacturer’s instructions [38]. The LNCaP cells (2.5 × 10^5^/mL) were seeded 48 hours before the experiments in a 24-well plate. The chalcone (100 μM) with or without TRAIL (100 ng/mL) was added to the cancer cells, and 48 hours later, the cells were washed with PBS and detached from the cell culture wells by trypsin. The cells were incubated in the dark with DePsipher solution at the concentration 5 μg/mL for 30 minutes at 37 ºC, then washed with reaction buffer with stabilizer, placed on a glass slide and covered with a glass coverslip. The stained cells were observed under a fluorescence inverted microscope IX51 (Olympus, Tokyo, Japan) using filter sets for FITC and TRITC. DePsipher (5,5’,6,6’-tetrachloro-1,1’,3,3’-tetraethylbenzimidazolyl carbocyanin iodide) is a fluorescent cationic dye which is used as a mitochondrial activity marker and detects early apoptosis in cells. DePsipher exhibits potential-dependent accumulation in mitochondria, indicated by a fluorescence emission shift from red (590 nm) to green (530 nm). In healthy cells, the mitochondria contain red spots following aggregation of the DePsipher within the mitochondria. In cells with disrupted potential the dye remains in its monomeric form in the cytoplasm and appears as entirely green fluorescence. 

### 3.8. Statistical analysis

The results are expressed as means ±S.D. obtained from three separate experiments performed in quadruplicate (n = 12) for cytotoxicity or duplicate (n = 6) for apoptosis. Statistical significance was evaluated using Bartlett Chi^2^ or Levene test followed by analysis of ANOVA. A p-values < 0.05 were considered significant.

## 4. Conclusions

We investigated the apoptotic and cytotoxic effects of chalcones and dihydrochalcones on prostate cancer cells in combination with TRAIL. Our findings indicated that all tested compounds: chalcone, 2’,6’-dihydroxy-4’-methoxychalcone, 2’,6’-dihydroxy-4’-methoxydihydrochalcone, 2’,6’-dihydroxy-4,4’-dimethoxydihydrochalcone and phloretin (4,2’,4’,6’-tetrahydroxydihydrochalcone) markedly augment TRAIL mediated apoptosis in LNCaP cells. Sensitization of prostate cancer cells to TRAIL-mediated apoptosis by chalcones and dihydrochalcones suggest the potential role of these compounds in anticancer immune defense in which endogenous TRAIL takes part. The TRAIL-mediated cytotoxic and apoptotic pathways may be a target of the chemopreventive agents in prostate cancer cells and the overcoming TRAIL-resistance by chalcones and dihydrochalcones may be one of the mechanisms responsible for their cancer preventive effects.
